# Adenocarcinoma in a Female Urethral Diverticulum With Gastric Differentiation: A Case Report

**DOI:** 10.7759/cureus.70203

**Published:** 2024-09-25

**Authors:** Samantha Johnson, Caitlin Turner, Hannah Lachmayr, Wylly Killorin

**Affiliations:** 1 Medical School, Mercer University School of Medicine, Columbus, USA; 2 Medical School, Mercer University School of Medicine, Savannah, USA; 3 Department of Urology, St. Francis Hospital, Columbus, USA

**Keywords:** oncologic urology, oncology, urethral neoplasms, urogenital neoplasms, urogenital pathology, urologic neoplasms, urology

## Abstract

This report describes a novel case of urethral adenocarcinoma in situ with enteric (gastric-type) differentiation in a 74-year-old female presenting with recurrent urinary tract infections and obstructive urinary symptoms. Magnetic resonance imaging (MRI) revealed a urethral diverticulum (UD), and a diverticulectomy was performed. Pathology from samples obtained during this operation revealed this rare subtype of malignancy. Our case highlights the importance of considering a broad differential diagnosis, even in patients with atypical presentations, and the need for further research to optimize the detection and treatment of this disease.

## Introduction

A urethral diverticulum (UD) is an abnormal outpouching of the urethral mucosa that arises from a defect in the fascial layers of the urethropelvic ligament [1]. UD is an uncommon condition that affects 1-6% of adult women [2,3] and typically presents in the third to seventh decade of life [4-6]. The classic presentation includes dysuria, dyspareunia, and post-void dribbling. Other associated symptoms include obstructive symptoms and recurrent urinary tract infections (UTIs) [7-10]. There are less than 130 reported cases of carcinoma associated with UD, with adenocarcinoma being the most frequent type of malignancy reported [11-14]. Although intestinal-type metaplasia is a well-documented phenomenon in the gastrointestinal system, it is rarely associated with the urinary tract [15]. In this study, we report a 74-year-old woman with recurrent UTIs and progressive obstructive urinary symptoms secondary to UD. Surgical management was pursued, and pathology revealed urinary adenocarcinoma in situ with enteric, gastric-type differentiation. 

## Case presentation

**Figure 1:**
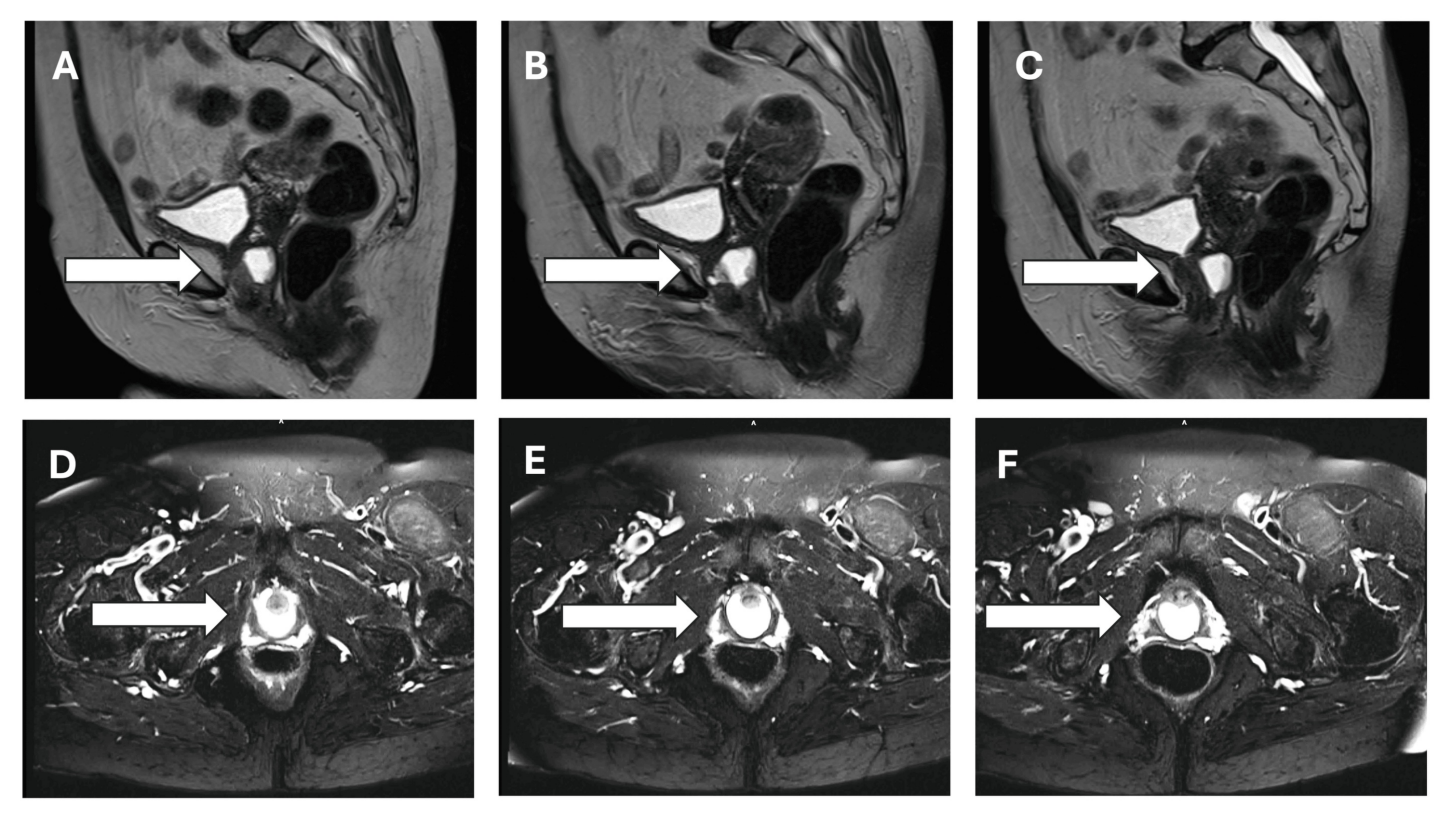
Sagittal (A-C) and axial (D-F) views of the urethral diverticulum T2-weighted magnetic resonance imaging of the urethra after 8 mL of intravenous contrast was injected. A 2.0 × 2.5 × 2.0 cm periurethral diverticulum (white arrow) with a small fluid level and mild thin internal stranding was noted. The white arrows point to the filling of the urethral diverticulum with contrast at progressive time points during the scan. A mass effect was noted from the diverticulum anteriorly.

A 74-year-old female presented with a history of recurrent UTIs, progressive urinary symptoms, and a UD. The patient reported occasional dysuria, frequency, and urinary urgency occurring over the past two years. The patient also had multiple afebrile UTIs with urine cultures positive for both methicillin-resistant *Staphylococcus aureus* and *Citrobacter koseri* during this time. The patient was planning to undergo a hysterectomy due to uterine fibroids and requested a urethral diverticulectomy to occur during the same operation. The physical examination of the patient under a valve and with a speculum revealed a UD that resulted in urinary leakage with applied pressure. A cystoscopy obtained after this exam could not directly visualize the UD. A magnetic resonance imaging (MRI) with and without contrast was ordered to further evaluate the patient, and a periurethral diverticulum with a small fluid level and mild thin internal stranding was revealed (Figure 1). This was noted to likely have occurred secondary to hemorrhage or prior infection. A urinalysis and a urine culture were obtained and were within normal limits and without bacterial growth noted, respectively.

**Figure 2:**
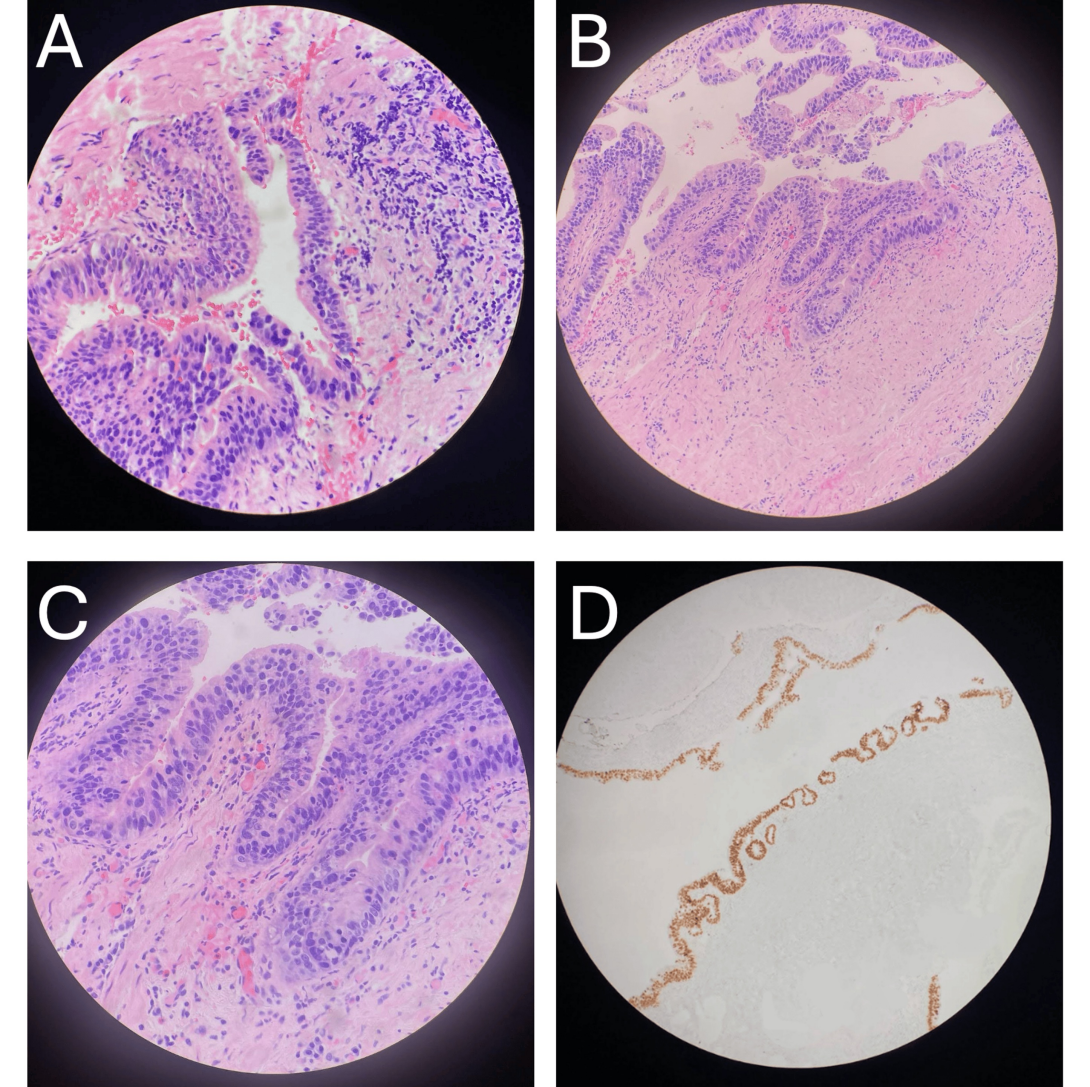
Histology of the urethral diverticulum consistent with a urinary adenocarcinoma in situ with enteric (gastric-type) differentiation (A-C) The epithelial lining of the urethral diverticulum composed of columnar epithelial cells resembling gastric foveolar epithelial cells, with foci of severe atypia/dysplasia and increased mitotic activity. A focal papillary formation is seen. (D) CDX-2-positive immunohistochemical staining of the urethral diverticulum is compatible with gastric-type differentiation of urinary adenocarcinoma in situ.

At this time, the patient was scheduled for a total robotic laparoscopic hysterectomy with bilateral salpingo-oophorectomy and a modified McCall’s culdoplasty with a transvaginal urethral diverticulectomy to follow. During the diverticulectomy, an incision was made to create an inverted U over the diverticulum, and a transverse incision was made through the periurethral fascia. The periurethral fascia was then carefully dissected off of a large diverticulum. The diverticulum was excised, and the incision was closed transversely. The operation was performed without complications, and a Foley catheter was placed, with the removal scheduled one week later. Subsequent pathology of the excised diverticulum revealed columnar-shaped epithelial cells, with many resembling gastric foveolar epithelial cells with severe dysplasia and increased mitotic activity. The specimen was also stained positive for CK7, CK20, GATA-3, and CDX-2; findings were compatible with a urinary adenocarcinoma in situ with enteric (gastric-type) differentiation (Figure 2). GATA-3 positivity correlated with small foci resembling cystitis cystica et glandularis as an incidental finding.

The follow-up discussion and referral were addressed at the two-week postoperative visit. The patient was referred to a local urologic oncologist for further evaluation. As the initial operation was not performed to excise adenocarcinoma and margins were not evaluated, the patient underwent re-excision of the diverticulum with the urologic oncologist. This secondary operation revealed no evidence of malignancy. The patient was evaluated four months postoperatively, and a follow-up cystoscopy demonstrated no evidence of disease within the bladder or the urethra. The patient has since been doing well, with improving urinary urgency and frequency noted. We will continue to follow up for surveillance. 

## Discussion

Urethral adenocarcinoma is a rare phenomenon, accounting for less than 0.02% of all cancer diagnoses in women [15]. We found that there are less than 130 reported cases of carcinoma associated with UD in the current literature [11-14]. Furthermore, we found no previously documented cases of urethral adenocarcinoma in situ with gastric differentiation. As our case involves incidentally discovering this rare malignancy, it is difficult to say whether guidelines for diagnosing urethral cancer would have been accurate. 

The American Urological Association (AUA) has recommendations for the diagnosis and treatment of urethral adenocarcinoma. However, for the initiation of this diagnostic workup to occur, one must have a strong clinical suspicion that a patient has such a malignancy. This relies heavily on a patient’s presenting symptoms, with the most common including dyspareunia, dysuria, and dribbling [16]. As seen in our case, there are many instances in which patients with atypical presentations may not warrant the routine ordering of guideline-recommended labs and imaging. These atypical presentations prompt us to examine further the adjustments that current guidelines may need to make to improve detection rates in this population.

Additionally, the procedures and imaging recommended by the AUA for the diagnosis of urethral neoplasms include obtaining a cystourethroscopy, MRI, and a positron emission tomography (PET) scan to evaluate and stage urethral neoplasms [16] that are not infallible. Our patient received an MRI prior to her urethral diverticulectomy, but no tumor was noted at that time. This is likely due to the in situ nature of the carcinoma. Because of this, there would be no reason to order additional testing as it would likely be deemed unnecessary, expensive, and time-consuming [1]. However, our patient may have benefited from further testing to avoid additional operations, thus reducing her risk of morbidity and mortality. This testing may have included urine cytology. This is something that is recommended following cystectomy for bladder cancer but has a limited role in the diagnosis of urethral carcinoma due to a sensitivity of less than 60% [16]. More studies are needed to determine the usefulness of urine cytology when urethral carcinoma is not detected on imaging but is possible given patient symptoms. 

The decision to perform a transvaginal diverticulectomy was made after careful consideration of the AUA recommendations for the treatment of a UD. As the transvaginal approach has an 86-98% cure rate [1], this was the agreed-upon surgical technique performed. Additionally, the inverted U-shaped incision with a transverse closure was performed during the operation, as this is the recommended approach to ensure that tension-free suture closure is obtained. This has been shown to preserve continence [1] and was therefore determined to be a beneficial undertaking based on our patient’s symptoms of urinary urgency. 

There are currently no guidelines for the timeline for surveillance after urethral diverticulectomy for the removal of urethral carcinoma. However, the guidelines for surveillance post-cystectomy for the treatment of non-metastatic bladder cancer can be adopted, as this monitors for recurrence in a retained urethra [16]. Therefore, a follow-up of every six to 12 months for two years can be followed unless a recurrence is found. The follow-up testing is also not mentioned in current AUA guidelines, but recommendations can be made if the adoption of the bladder cancer follow-up is used. This includes urine cytology at follow-up appointments, along with cystoscopic visualization [16]. As such, our patient will continue to be monitored at these time points. 

The fact remains that there is still much to learn about urethral cancer and its diagnosis and treatment. Treatment standards are based on factors such as tumor histology [16]. Since adenocarcinoma in a UD with enteric, gastric-type differentiation has never been reported, the most efficacious treatment regimens can only be theorized. Based on studies of non-differentiated adenocarcinoma, treatments such as the diverticulectomy performed on our patient are reasonable to pursue [16]. However, the likelihood of recurrence and the unprecedented subtype of genitourinary cancer require fervent surveillance to ensure any future recurrence is detected immediately. 

## Conclusions

Due to the low incidence of UD in females and low risk of carcinoma of the UD, suspicion for malignancy can be reasonably low in a patient presenting with recurrent UTIs and no classic signs of malignancy. This case demonstrates the importance of a multi-disciplinary (i.e., urology, oncology, and pathology) approach in a patient’s diagnosis and care. The case also emphasizes the utility that the multimodal diagnostic approach seen in the AUA guidelines (e.g., cystourethroscopy, MRI, and PET scan) may provide. While the current literature does not offer definitive guidelines for the diagnosis and treatment of adenocarcinoma in a female UD with gastric differentiation, we can look to urethral adenocarcinoma and UD recommendations to guide future plans. Further research on the combination of diagnostic modalities to enhance the sensitivity of urethral carcinoma detection while decreasing the rate of false negatives is a promising avenue of future focus. Additionally, research studies focusing on the similarities and differences between urethral carcinoma cases would be helpful in expanding the current understanding of risk factors, subtypes, and treatment responses in these patient populations. By focusing on advancing our knowledge of this rare disease, we will be able to improve patient outcomes while reducing morbidity and mortality rates in a previously unstudied population.
